# The Outside World

**Published:** 1897-11

**Authors:** 


### THE OUTSIDE WORLD.

Before the Mayors' Convention at Columbus, Ohio, in September, Mr. Nathan Strauss, of New York, said: "There is practically no milk delivered for general consumption in cities that is fit to be fed in its natural state to young children. I think I have demonstrated the proposition that many thousands of infants' lives are annually sacrificed by neglect to supply, for the nutriment of children, milk which has been sterilized. I hold that neglect to be criminal, and I leave it to you to fix the responsibility for it. We punish murder with the penalty of death, and yet we allow murder to be committed by the wholesale in every populous community of this land with no thought of its punishment and little thought of its prevention. You have the means under your control by which these babies can be saved. I ask you, Will you not apply them? Men are found capable of acts of heroism in presence of danger less threatening and less surely fatal. All that I plead for is the extension of the activity of local boards of health into a sphere which is legitimately theirs, but which they have so far lacked the conviction and courage to occupy."

From the far West comes the report that Dr. T. B. Short, of Chehelis, Wash., who was recently called to the stable of J. B. Rice to examine a sick mule, contracted glanders, and died of its effects. A hired man on the place of Mr. Rice has also succumbed to the disease, and as several others were exposed to the disease through the mule it is feared they may be affected also.

Janie T., the two-year-old trotter, making the pace in 2.15¼, breaks the record of the two-year-olds, and lowers the best filly-record one-half second. Such are the wonderful strides in raising and training the American trotting-bred horses.

The new team record made by John R. Gentry and Robert J. at Glen Falls, N. Y., on October 8, 1897, places it at 2.08.

The *Blue Book* is nearing completion, and will emerge from the printer's hands early in November, much enlarged and improved as a result of the delays.

The school of farriery for Greater New York, under the promotion of Prof. R. S. Huidekoper, still keeps on apace, and it is hoped to get it under full force this winter.

The demand for choice horses is not yet ended. Mr. Belmont's recent sale of his seventeen carriage horses at an avearge of over one thousand dollars each must be felt as an indication that there will never be an oversupply of good carriage horses.

Philadelphia has now among its prominent large buildings a professional one where every possible want is provided for the several branches of medicine, and where the privacy of a medical man's office may still be maintained.

Oil of sassafras one part, alcohol five parts, will keep mosquitos away.

A German writer strongly recommends ichthyol for insect-bites or stings, used in its pure state, applied directly to the parts affected.

*Modern Medicine and Bacteriological Review* quotes a French authority as conducting bacteriological experiments as to the existence of tuberculosis among fish. Carp placed under favorable environments developed tuberculosis in aggravated form.

President Purcell of the National Horseshoers' Association, recommends the establishment of a National School of Farriery.

Milking-machines are rapidly being brought to a practical and perfect production as to promise being a strong factor in increasing the number of dairy-cows throughout the West.

An advocate of dehorning says nervousness and dread among dairy-cows do not induce increased milkgiving. Quietness and gentleness should reign supreme on the dairy-farm.

The value of our range-horses for cavalry use is only slightly appreciated. Their powers of endurance are phenomenal, under great hardships of poor food and irregular watering and little care.

The horses of the street-cleaning department of Greater New York are used without check-reins.

Nebraska loses one of her pioneer and foremost horsemen in the death of Dr. A. O. Halliday, of Brownville. The trotter and pacer were his favored kind.

Mr. C. E. Stubbs, of Denver, Colorado, will look after American equine interests abroad, as special commissioner, studying for our horse-breeders the types of cavalry, carriage, and general purpose horses desired for the foreign markets.

The *Medical Fortnightly*, in an interesting article termed "Chinese Doctors and Their Practice of Medicine," records some curious remedies as in use in medical practice: To eat the flesh of a white horse with a black head will cause insanity. Ordinary horse-flesh may be eaten with impunity, excepting parts covered by the saddle, and the liver. Excrement of a cat is advised for asthma, as a close relation is believed in between asthma and purring. For dyspepsia yellow-dog meat is recommended, and black-dog meat for kidney diseases. Donkey and white-horse urine are used for worms, loss of appetite, and obstinate vomiting. Feline placentæ are given in consumption.

